# Copper and Zinc Interactions with Cellular Prion Proteins Change Solubility of Full-Length Glycosylated Isoforms and Induce the Occurrence of Heterogeneous Phenotypes

**DOI:** 10.1371/journal.pone.0153931

**Published:** 2016-04-19

**Authors:** Svetlana Brim, Martin H. Groschup, Thorsten Kuczius

**Affiliations:** 1 Institute for Hygiene, University of Münster, Robert Koch-Strasse 41, 48149 Münster, Germany; 2 Friedrich-Loeffler-Institute (FLI), Institute for Novel and Emerging Infectious Diseases, Boddenblick 5a, 17493 Greifswald, Insel Riems, Germany; University of Maryland School of Medicine, UNITED STATES

## Abstract

Prion diseases are characterized biochemically by protein aggregation of infectious prion isoforms (PrP^Sc^), which result from the conformational conversion of physiological prion proteins (PrP^C^). PrP^C^ are variable post-translationally modified glycoproteins, which exist as full length and as aminoterminally truncated glycosylated proteins and which exhibit differential detergent solubility. This implicates the presence of heterogeneous phenotypes, which overlap as protein complexes at the same molecular masses. Although the biological function of PrP^C^ is still enigmatic, evidence reveals that PrP^C^ exhibits metal-binding properties, which result in structural changes and decreased solubility. In this study, we analyzed the yield of PrP^C^ metal binding affiliated with low solubility and changes in protein banding patterns. By implementing a high-speed centrifugation step, the interaction of zinc ions with PrP^C^ was shown to generate large quantities of proteins with low solubility, consisting mainly of full-length glycosylated PrP^C^; whereas unglycosylated PrP^C^ remained in the supernatants as well as truncated glycosylated proteins which lack of octarepeat sequence necessary for metal binding. This effect was considerably lower when PrP^C^ interacted with copper ions; the presence of other metals tested exhibited no effect under these conditions. The binding of zinc and copper to PrP^C^ demonstrated differentially soluble protein yields within distinct PrP^C^ subtypes. PrP^C^–Zn^2+^-interaction may provide a means to differentiate glycosylated and unglycosylated subtypes and offers detailed analysis of metal-bound and metal-free protein conversion assays.

## Introduction

Prion diseases are fatal neurodegenerative disorders characterized clinically by a long incubation period followed by a rapid course of disease, and biochemically by the accumulation of the infectious prion protein PrP^Sc^. PrP^Sc^ originates from a host encoded prion protein (PrP^C^) by conformational conversion. As the mechanism of the folding of PrP^Sc^ is not yet clear, the conversion is associated with dramatic changes in biochemical and biophysical properties. PrP^C^ is sensitive to proteolysis, is completely hydrolysed, and has high α-helix content [1]; whereas PrP^Sc^ demonstrates an increase of β-sheet structures [2] leading to hydrophobicity, the formation of fibrillar depositions and partial protease resistance. Following expression, PrP^C^ is post-translationally modified by the formation of a glycophosphatidyl-inositol (GPI) anchor and a disulphide bond. Glycans attach to one or two asparagine residues yielding in di-, mono- and unglycosylated proteins [3].

PrP^C^ proteins are characterized by heterogeneous phenotypes in distinct brain regions and display several subtypes, which overlap distinct protein patterns identified by means of differential detergent solubility [4–5]. The differential glycoprotein pattern observed may be a result of distinct biological functions such as synaptic transmissions, transport processes and metal binding, indicating an involvement in neuroprotective and oxidative stress reactions [6–9]. PrP^C^ is known to be a metalloprotein having the capability of binding multiple zinc and copper ions that stimulate the endocytosis of PrP. Additionally, the protein is thought to be associated with metal-dependent enzymatic functions and with copper homeostasis [10–15]. A highly conserved octarepeat region is located within the aminoterminal region containing identical repeats (PHGGGWGQ) that were shown to have a high affinity for copper [16]. For each of the histidine residues within the octarepeats, one copper ion can be bound with a higher affinity than other divalent ions [17–18]. Both copper and zinc binding impart conformational changes in the structure of PrP demonstrated by the formation of protease resistance and protein insolubility [19–20]. Additional, full-length PrP^C^ can be truncated at the aminoterminus under physiological conditions generating a glycosylated C1 fragment which is present in brains in substantial amounts [21].

The post-translational modifications themselves and the structural changes due to metal ion interactions increase the variability of PrP^C^ proteins. It is not known whether the modifications, the impact of metal binding or both are essential for the development of prion diseases. In comparison to the incidence of various existing PrP^C^ types expressed in normal tissue and brain, very few PrP^Sc^ types have been identified in diseased species. This suggests that different PrP^C^ isoforms may vary in their potential for conformational conversion. To reduce the conversion efficiency to PrP^Sc^, it is important to first identify and target PrP^C^ subtypes with a high-yield conversion. In this study, we performed metal-binding analyses on phenotypes of heterogeneous brain PrP^C^ isoforms derived from uninfected humans, bovine, sheep and mice in order to identify protein subtypes with either high or low solubility. Our results reveal that PrP^C^ markedly exhibited a lower solubility when zinc was bound to the protein, whereas copper binding showed little effect on solubility. Differential solubility as a result of metal binding offers a new tool to examine PrP^C^ isoforms bound to protein complexes.

## Materials and Methods

### Antibodies

Monoclonal anti-PrP antibodies (mabs) SAF34 and SAF70 were prepared by immunizing knock-out mice with formic acid-denatured, hamster scrapie-associated fibrils (263K) [22]. Mab SAF34 recognizes an epitope in the octarepeats detecting the aminoterminal sequence, whereas mab SAF70 binds to the core protein at amino acids 156–162. The antibodies in this study were applied as ascitic fluids obtained from mice and originated from one slot. The brain marker neuron specific enolase (NSE) was used as a control protein detected by using a monoclonal mouse anti-NSE antibody, which was purchased as a purified IgG antibody (Dianova, Hamburg, Germany).

### Brain tissue

Brain tissue was obtained from TSE-free humans, sheep, bovine and mice. Human brain tissue derived from five individuals was pooled from several unspecified sample regions and comprised mostly of cortex and cerebellum. All experiments on human subjects were conducted in accordance with the Declaration of Helsinki and all procedures were carried out with the adequate understanding and written consent of the subjects. Brain tissue samples were obtained from the Brain Bank, Institute of Neuropathology at the University Hospital Münster [4]. Consent for autopsy and scientific examination was obtained from the legal representatives (Ethics Committee of the Westphalia Chamber of Physicians and the Faculty of Medicine, University of Münster) for all subjects in accordance with the local institutional review board. The bovine and sheep brain samples were provided from the Chemische und Veterinäruntersuchungsamt Münsterland—Emscher—Lippe after routine analyses for TSE and slaughtering processes in slaugtherhouses in Northrhine Westfalia, Germany, and homogenates were prepared from different brain areas in four biological replicates. Mouse brain tissue was obtained from C57BL wild-type mice and T182N transgenic mice, which are characterized by a deletion of the glycosylation site at codon 182 via an amino acid exchange from asparagine (N) to threonine (T) [23]. Animal experiments were approved, and their implementation supervised by the competent authorities of the Federal State of Mecklenburg-Western-Pomerania, according to the German Animal Welfare Act. The experiments in mice described in this manuscript were approved by the competent authority of the Federal State of Baden-Wuerttemberg, Germany on the basis of national and European legislation, namely the EU council directive 86/609/EEC for the protection of animals used for experiments (Regierungspräsidium Tübingen BFAV-Tierversuchsvorhaben Nr. 156—Herstellung und Charakterisierung transgener Mauslinien mit gewebespezifischer Expression des Prionproteins oder der Expression artifizieller Prionproteine). Breeding occurred at the Federal Research Centre for Virus Diseases of Animals, Tübingen, Germany, according to the rules and conditions listed above. Mice obtained from the mouse breeding facility of the Friedrich-Loeffler Institute were fed ad libitum with commercial mouse feed and were euthanized by carbon dioxide intoxication. Animals were kept in Macrolon cages (type 2 long; groups of maximum 5 mice respectively) in rooms in which a natural day/night light cycle regime was run, and they were controlled daily for their clinical status. Four whole brains from both wild-type and transgenic mice were pooled separately for homogenization.

### Homogenate preparations

Brain tissues were weighed and suspended in nine volumes of Tris buffered saline (TBS; pH 7.4) in order to prepare 10% (w/v) homogenates followed by the application of 2% N-octyl-β-D-glucopyranoside (OGP). Electric homogenizers were used for consistent homogenization followed by intensive ultrasonication as described [5]. Samples were stored at -20°C until use, and experiments were carried out with one charge of a pooled homogenate under standardized conditions.

### Addition of divalent cations, SDS and EDTA

Divalent cations such as CuCl_2_, ZnCl_2_, MnCl_2_, MgCl_2_, NiSO_4_, CaCl_2_, and CoCl_2_ (Sigma, Taufkirchen, Germany), each at a concentration of 1 mM or otherwise indicated, were added to the samples and incubated at room temperature for 30–60 min. To separate cellular prion protein fractions of high and low solubility, homogenates were centrifuged at 16,000 × *g* for 10 min resulting in supernatants and pellets. Pellets were washed with TBS to remove residual supernatant proteins and were resuspended in SDS-loading buffer.

To demonstrate the metal binding to PrP^C^, samples were incubated with the metal chelator ethylene diamine tetraacetic acid (EDTA; Serva, Heidelberg, Germany) or with sodium dodecylsulphate (SDS) in indicated concentrations at room temperature for 30 min, followed by centrifugation as described.

### Enzymatic treatment of protein samples

The enzyme bromelain was purchased from Sigma-Aldrich (Taufkirchen, Germany) as a chromatographically purified protease from pineapple stem. Protein suspensions from brain were treated enzymatically with bromelain at 37°C with gentle agitation using a concentration of 50 µg/ml for 60 min. Proteolysis was terminated by heating at 99°C for 10 min.

### Immunoblot analysis

Samples resuspended in SDS-loading buffer were denatured at 99°C for 5 min. Proteins were separated using sodium dodecyl sulphate polyacrylamide (13%) gel electrophoresis (SDS-PAGE) in a mini slab gel apparatus (Bio-Rad, Munich, Germany). After electrotransfer onto polyvinylidene difluoride (PVDF) membranes (Immobilon-P; Roth, Karlsruhe, Germany) using a semi-dry blotting system (Roth, Germany), non- specific binding was blocked on membranes by incubation in TBS containing 0.05% Tween 20 (TBST) and 1% (w/v) non-fat dry milk powder for 1 h at room temperature. Specific detection of PrP^C^ was accomplished by incubating the membranes with the antibodies indicated at room temperature over night. Membranes were washed with TBST prior to incubation with peroxidase-conjugated goat anti-mouse immunoglobulin antibodies (Dianova, Hamburg, Germany) as secondary antibody for 2 h at room temperature. After washing with TBST PrP^C^ bands were visualized using a chemiluminescence enhancement kit (Thermo Scientific, Bonn, Germany).

## Results

### Metal-binding to PrP^C^ results in a decrease of protein solubility

**Fig 1:**
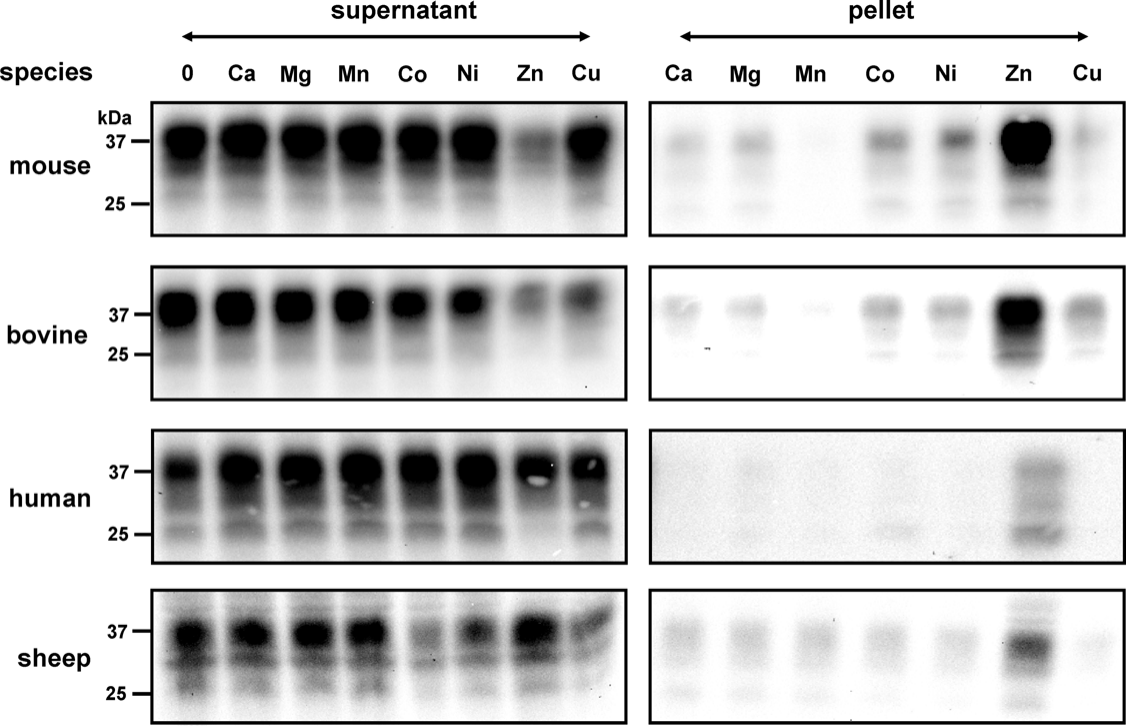
Interaction of metals with PrP^C^ induced a change to low solubility. Immunoblot analysis of brain homogenates (10%) derived from C57BL wild-type mice, bovine, human and sheep is as indicated. Homogenates were pre-incubated with various metal ions (1 mM) followed by centrifugation to generate a protein fraction of high solubility in the supernatant and of low solubility in the pellet. The pellet fractions were re-suspended in homogenate buffer back to the original volume. Following the addition of sample buffer, proteins were denatured by heating, and identical volumes were loaded for separation on SDS-PAGE and subsequent immunoblotting. PrP^C^ specific signals were detected on immunoblots by mab SAF34 and visualized using a chemiluminescence substrate. Unlike other metals, the interaction of ZnCl_2_ with PrP^C^ led to reduced protein solubility in samples from all four species.

Homogenized brain tissue suspensions from human, bovine, sheep and mouse were treated with the detergent N-octyl-β-D-glucopyranoside (OGP), which is non-ionic, and does not denature proteins. After centrifugation, the highly soluble proteins in the supernatants were exposed to various metal ions prior to incubation. Although we exclusively used proteins with high solubility, the binding of zinc to PrP^C^ induced a considerable shift of proteins into a PrP^C^ isoform of low solubility retrievable in the pellet after high-speed centrifugation (Fig 1). The incubation of PrP^C^ with copper ions clearly had a much lower effect on decreasing the solubility of PrP^C^ under these conditions; the levels of PrP^C^ with low solubility were only marginally higher than those observed in the presence of Co^2+^ and Ni^2+^. PrP^C^ remained highly soluble in the presence of Ca^2+^, Mg^2+^ and Mn^2+^ ions as well as in the absence of metals.

**Fig 2:**
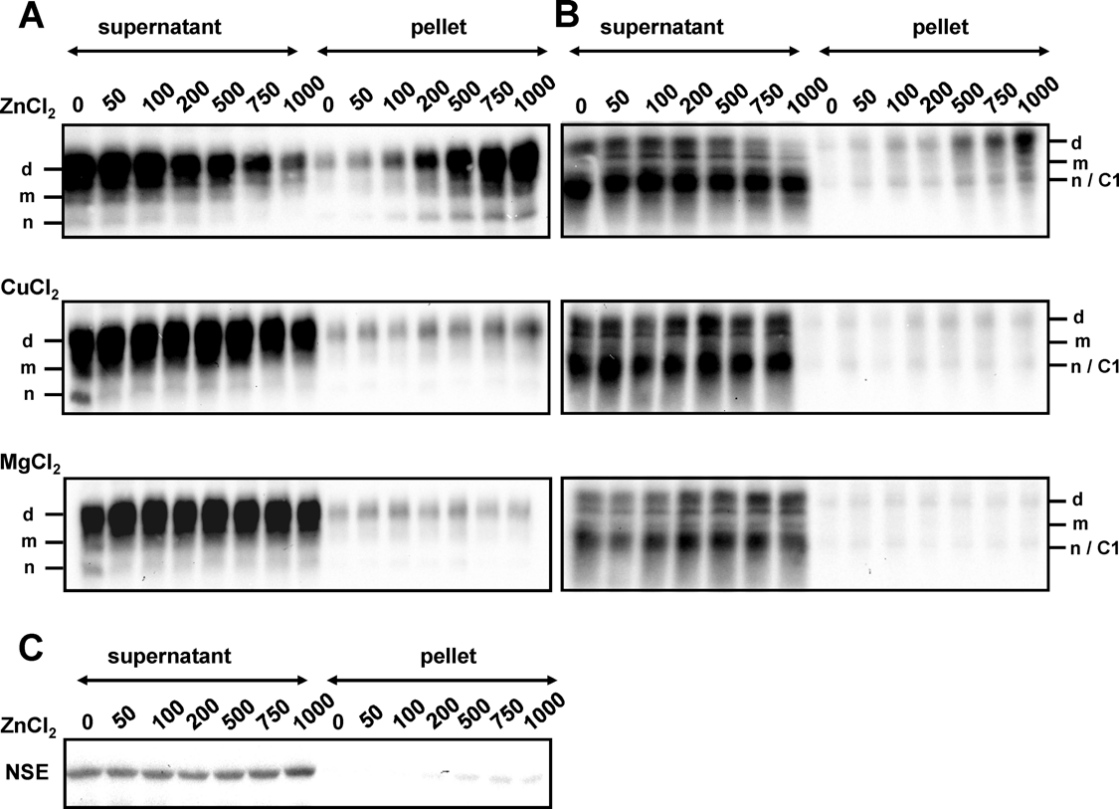
Reduced PrP^C^ solubility is an effect specific to the binding of certain metal ions. Protein suspensions from brain homogenates derived from C57BL wild-type mice were reacted with increasing concentrations of ZnCl_2_, CuCl_2_ and MgCl_2_ as indicated. Proteins were centrifuged resulting in a separation of PrP^C^ into a fraction of high solubility in the supernatant and a fraction of low solubility in the pellet. Following SDS-PAGE and immunoblotting, PrP^C^ signals were visualized using mabs SAF34 (A) and SAF70 (B) followed by chemiluminescence substrate development. PrP^C^ specific bands are indicated with d (diglycosylated), m (monoglycosylated), n (nonglycosylated) and C1 (truncated glycosylated C1 fragment). Neuron-specific enolase (NSE) was used as control (C).

To verify a zinc specific effect, mouse PrP^C^ was incubated with increasing concentrations of the zinc ion (Fig 2). We found that ZnCl_2_ reduced PrP^C^ solubility at concentrations as low as 50 to 100 µM, and insolubility rose as Zn^2+^ concentrations were increased. With respect to the protein profile in the pellet fraction, the full length diglycosylated isoforms dominated, whereas the truncated glycosylated C1 proteins and the unglycosylated isoforms exhibited lower intensities. Zn^2+^ bound PrP^C^ was determined by using the monoclonal antibody SAF34 that recognizes an epitope within the octarepeats (Fig 2A). To analyze whether this banding profile originated from an antibody specific effect, we examined protein profiles by binding mab SAF70 to the core protein region (Fig 2B). Interestingly, two different banding patterns with Zn^2+^ interaction were revealed. In the supernatants, a dominant band was observed, which consisted of highly soluble PrP^C^ having a molecular mass of unglycosylated PrP^C^. This protein signal resulted from an overlay of unglycosylated PrP^C^ and truncated glycosylated C1 protein, because both isoforms had the same electrophoretic mobility. Unglycosylated PrP^C^ demonstrated lower signal intensity than truncated glycosylated C1 protein shown by protein deglycosylation using N-glycosidase F treatment (data not shown). In contrast, low PrP^C^ signals were observed for the full-length glycosylated isoforms. In the pellets, however, high signals were detected for the full-length diglycosylated PrP^C^ band; these intensified as Zn^2+^ concentrations were increased. This result indicated that binding of Zn^2+^ to PrP^C^ is directly correlated to the solubility reduction of the full-length diglycosylated form of PrP^C^. This differential solubility effect as a result of Zn^2+^-PrP^C^ interaction was not observed with Cu^2+^ and Mg^2+^ under the same conditions and specificity was demonstrated by using the neuron specific enolase (NSE) as a control protein, which remained highly soluble within the range of Zn^2+^concentration used (Fig 2C).

### Glycosylated PrP^C^ isoforms interact with zinc to lower the solubility of proteins

**Fig 3:**
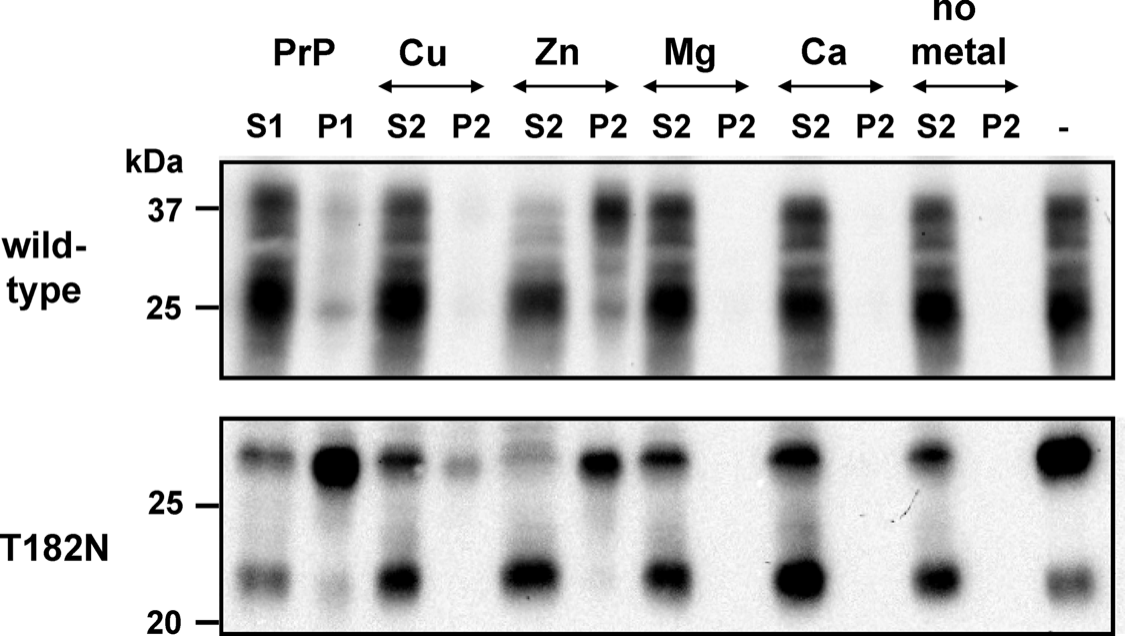
The glycosylation states influenced changes in solubility of mouse prion isoforms as a result of metal-binding. Pooled mouse brains from C57BL wild-type and transgenic T182N mice were homogenized (10%) and supplemented with 2% N-octyl-β-D-glucopyranoside (OGP) followed by centrifugation to obtain supernatant (S1) and sedimented protein (P1). Highly soluble proteins from the S1 fraction were pre-incubated in the absence or presence of metal ions such as CuCl_2_ (Cu), ZnCl_2_ (Zn), MgCl_2_ (Mg) and CaCl_2_ (Ca), 1 mM each. Proteins were once again separated by centrifugation into fractions of high and low solubility represented as supernatants (S2) and pellets (P2), respectively. After immunoblotting, PrP^C^ proteins were identified using mab SAF70 and signals were visualized by chemiluminescence substrate development. Glyosylated full-length PrP^C^ proteins of both mouse types were detected in the pellet fraction when bound to zinc ions. A considerably lower effect was observed in the T182N PrP^C^ interaction with copper ions, whereas other metals played no role in structural changes resulting in sedimentation. When metal ions were bound, mainly full length glycosylated PrP^C^ changed into the pellet fraction.

To analyze the influence of the glycosylation grade on the increase of pelleted PrP^C^-Zn^2+^ we added metal ions to wild-type mouse brain homogenates featuring two glycosylation sites, which can be modified at one or both sites and to mouse homogenates of the transgenic mouse line T182N characterized by one deleted glycosylation site [22] (Fig 3). In immunoblots the monoglycosylated band was observed as a strong signal, whereas the signal at the molecular mass of the unglycosylated PrP^C^ was hardly visible. Additionally, an unknown fragment derived from the carboxy terminus was detected at approximately 22 kDa.

High signal intensities of full-length diglycosylated wild-type PrP^C^ bound to Zn^2+^ were detected in the pellet fraction, whereas glycosylated C1 fragment and unglycosylated PrP^C^ was abundant in the supernatant (Fig 3). In contrast, very faint signals of all PrP^C^ isoforms were observed in the pellet with copper interaction and no signals were detected with manganese and calcium or as well as in the absence of metals. PrP^C^ derived from transgenic mice T182N exhibited a lower solubility than fully glycosylated wild-type PrP^C^. However, Zn^2+^ triggered a decrease in the solubility of the full-length glycosylated isoform in proteins from both T182N as well as wild-type mice. This finding indicates that zinc binding results in an important structural change in glycosylated PrP^C^. Under these conditions, a weak yet visible effect was also seen with Cu^2+^. The unknown 22 kDa fragment of T182N was always detected in the highly soluble fraction.

### Decreases in PrP^C^ solubility as a result of metal binding is a reversible process

**Fig 4:**
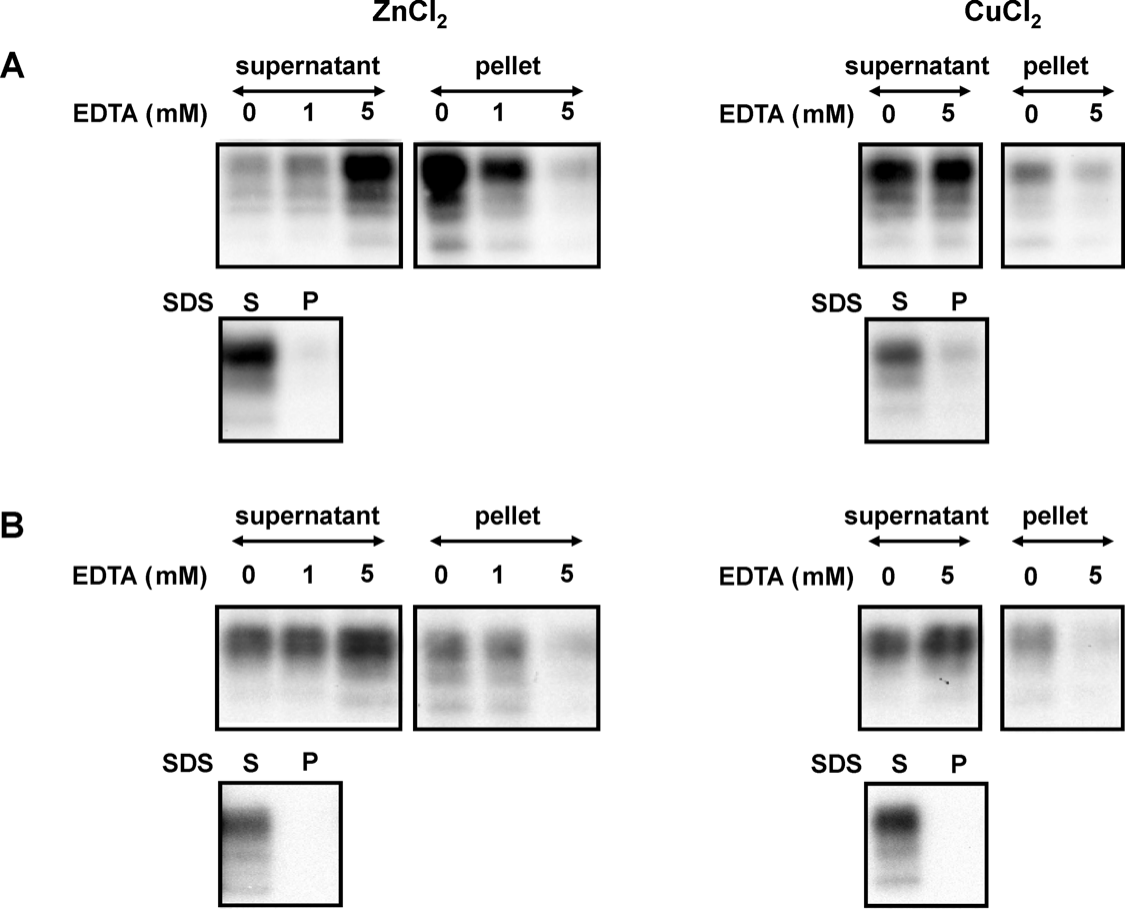
Reduced solubility due to ZnCl_2_ and CuCl_2_ is reversed by EDTA and SDS application. Pooled brain tissues derived from C57BL wild-type mice (A) and bovine (B) homogenized in TBS and 2% N-octyl-β-D-glucopyranoside were supplemented with ZnCl_2_ (1 mM) and CuCl_2_ (1 mM) followed by incubation in the absence (0) or presence of EDTA in concentrations indicated or 1% SDS. Following centrifugation, proteins were separated into fractions of high solubility in the supernatant and low solubility in the pellet. PrP^C^ signals were detected using mab SAF34 and visualized by chemiluminescence substrate development.

The effect of PrP^C^ interaction with zinc, which results in a decrease in solubility, was reversible by treatment with ethylene diamine tetraacetic acid (EDTA) and sodium dodecylsulphate (SDS) (Fig 4). Mouse and bovine homogenates pre-incubated with zinc or copper ions were treated with the metal chelator EDTA. EDTA at equimolar and higher concentrations reversed PrP^C^ from low to high solubility. Reversibility was determined using the amino terminal region-binding antibody SAF34 and the core region-binding antibody SAF70 as well (not shown). High solubility of zinc and copper-bound PrP^C^ was also induced by treatment with the protein denaturing detergent sodium dodecylsulphate (SDS).

### Truncation of the PrP^C^ amino-terminus prior to metal binding prevented the shift to low solubility

**Fig 5:**
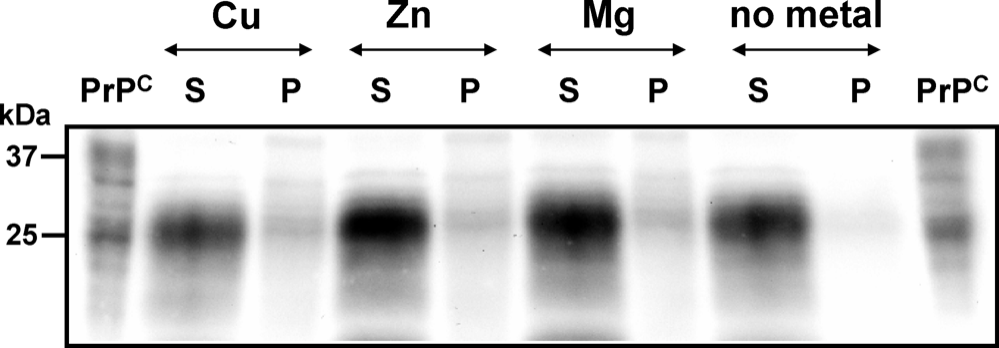
Amino-terminally truncated PrP^C^ isoforms are unable to bind metal ion. Proteins from pooled wild-type C57BL mouse brain homogenate were treated enzymatically with bromelain (50 µg/ml, 37°C, 60 min) generating a carboxy-terminal core protein lacking the octapeptide region to which divalent cations might bind. After incubation in the presence of CuCl_2_ (Cu), ZnCl_2_ (Zn) and MgCl_2_ (Mg) in concentrations of 1 mM each or in the absence of metal ions (no metal), proteins were separated by centrifugation into fractions of high and low solubility, respectively, represented as supernatants (S) and pellets (P). Proteins were separated by SDS-PAGE, immunoblotted and PrP^C^ signals were visualized using mab SAF70 and chemiluminescence substrate development. Untreated protein (PrP^C^) was used as control.

Metal ions bind within the octarepeats at histidine residues with high affinity, whereas zinc binds with lower affinity [18;24]. To verify that the PrP^C^-Zn^2+^ phenotype is the result of metal binding to the octarepeats, we partially truncated the full-length protein by enzymatic treatment with bromelain obtained from *Ananas comosus*. The amino-terminal region was cleaved off, whereas a stable core protein consisting of glycosylated and nonglycosylated isoforms was generated (Fig 5). Removal of the amino-terminus prior to incubation in the presence of metals prevented PrP^C^ from changing to low solubility. This result indicates an interaction of Zn^2+^ with the N-terminal region of PrP^C^.

### PrP^C^ solubility by combined and serial metal incubations

**Fig 6:**
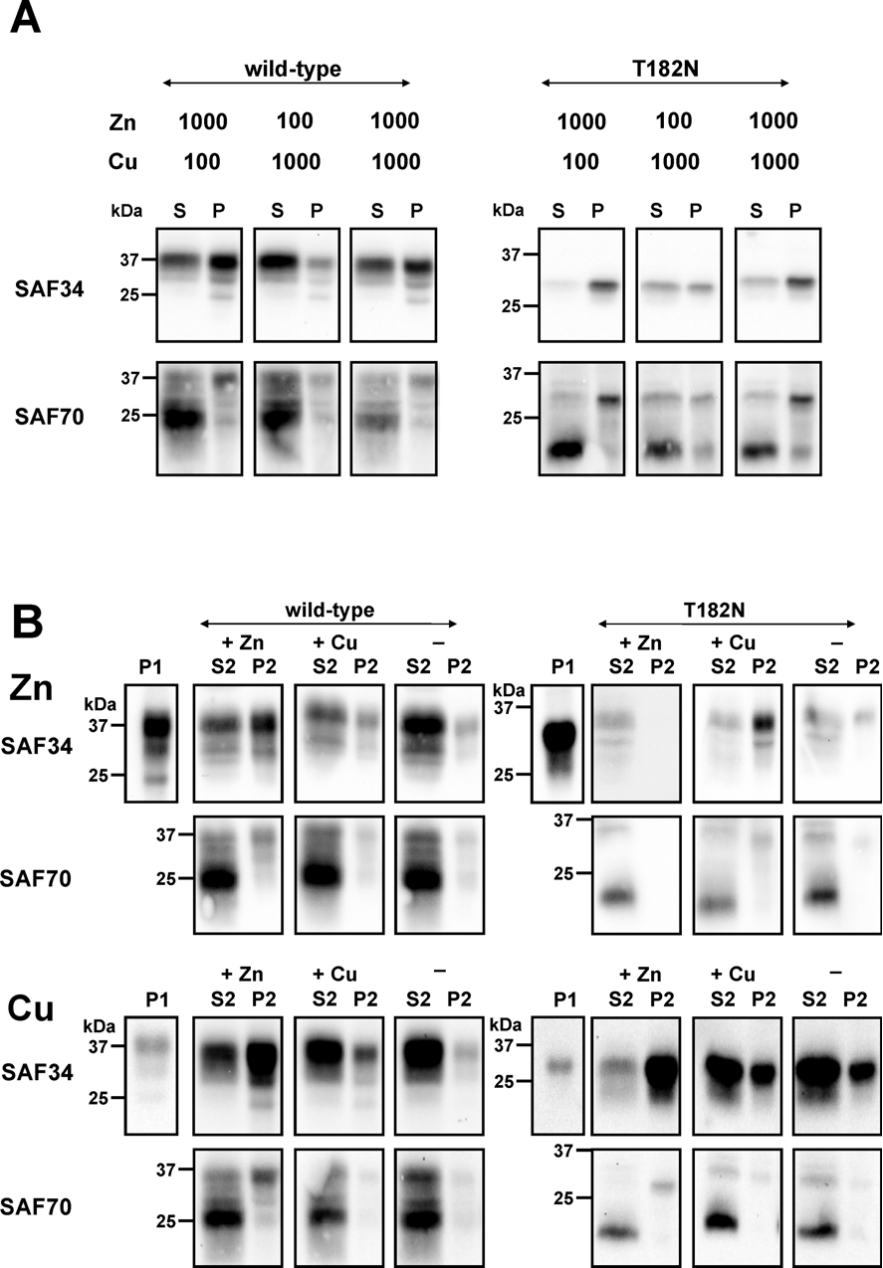
The PrP^C^–Zn^2+^ phenotype dominated in PrP^C^ interactions after simultaneous and serial CuCl_2_ and ZnCl_2_ incubation. (A) Brain homogenate proteins (10%) derived from the C57BL wild-type and T182N transgenic mice were suspended in TBS with OGP (2%) followed by simultaneous incubation with ZnCl_2_ (Zn) and CuCl_2_ (Cu) in concentrations of 100 and 1000 µM as indicated. Proteins were separated by centrifugation into a protein fraction of high solubility represented as supernatant (S) and low solubility PrP^C^ in the pellet (P). (B)Aliquots of highly soluble proteins from C57BL wild-type and T182N transgenic mice were pre-treated with zinc (Zn) and copper (Cu) ions (1 mM) each. As a result of centrifugation, proteins were separated into a pellet fraction (P1) and into highly soluble protein fractions, which were incubated additionally with ZnCl_2_ (+ Zn) and CuCl_2_ (+ Cu; 1 mM each) or in the absence of ions (-) as controls. Highly soluble proteins were detected in the supernatant S2, whereas poorly soluble PrP^C^ were detected in the pellet P2 after centrifugation. Copper ions were unable to alter the PrP^C^–Zn^2+^ phenotype, which would have shown a conversion of pelleted PrP^C^ to a fraction of highly soluble proteins. Protein suspensions were separated by SDS-PAGE, immunoblotted and PrP^C^ were detected using mabs SAF34 and SAF70 as indicated. Signals were visualized after chemiluminescence reaction on an imager.

In the next series of experiments, we analysed the solubility of PrP^C^ following combined and serial incubations with copper and zinc ions (Fig 6). The Zn^2+^-PrP^C^-phenotype predominated, even following simultaneous incubation with both copper and zinc ions in identical concentrations (Fig 6A). The three isoforms—un-, mono- and diglycosylated PrP^C^—were abundant in the pellet fraction when using mab SAF34 as a detection antibody. The full-length diglycosylated PrP^C^ from wild-type mice and the full-length monoglycosylated derived from the mutant T182N mice showed high signal intensity in the pellet, whereas the truncated glycosylated C1 fragment and the unglycosylated wild-type PrP^C^ isoforms dominated the supernatant when using mab SAF70. This Zn^2+^-PrP^C^-phenotype persisted as well in samples that had been treated serially with different metal ions (Fig 6B). Using the antibodies mab SAF34 and mab SAF70, an increase in the abundance of copper loaded PrP^C^ was observed in the pellets after Zn^2+^ ions had been independently added to the proteins. Collectively, zinc binding to PrP^C^ had an important influence on decreasing the solubility of PrP^C^, whereas copper binding failed to neutralize the Zn^2+^-PrP^C^-phenotype. This suggests an additional metal-protein interaction and a different biochemical characteristic independent of the copper-PrP^C^ interactions.

## Discussion

Although the biological functions of PrP^C^ remain an enigma, it is known that PrP^C^ interacts with several metal ions such as Cu^2+^, Zn^2+^, Mn^2+^, Pd^2+^, Pt^2+^ and Co^2+^ when sufficient concentrations are present [25–30]; these metallochemical changes might facilitate protein conversion leading to the development of prion diseases [31]. The binding of Zn^2+^ and Cu^2+^ are accompanied by structural changes and decreased solubility [19–20]. However, the effect of metal binding as a means of protein differentiation has not yet been analyzed. In the present study, we distinguished PrP^C^ isoforms using high-speed centrifugation in conjunction with metal binding. The interaction of zinc with PrP^C^ induced an important shift from high to low protein solubility, mainly affecting the full-length glycosylated isoforms. Although both metals bound to the amino-terminal region, copper binding did not reveal the structural changes affiliated with low solubility under these conditions. Copper binds to histidine residues via the octarepeats and to single sites outside this region with high efficiency with a K_d_ of 6.7 to 14 µM [16–18;24;32–33]. Zinc interacts with a lower affinity than copper, and the K_d_ was determined as approximately 200 µM [29].

The interaction between PrP proteins seems to be mediated by copper and zinc binding within the amino-terminal region of PrP [34], which could influence differential solubility. It is conceivable that Cu^2+^ acts as a bridge between PrP proteins [35–36]. Such interactions are promoted by copper and zinc [37], with Zn^2+^ having a greater influence on these interactions than Cu^2+^. This Zn^2+^ induced PrP-PrP interaction may have decreased protein solubility concurrent with the level of Zn^2+^ bound PrP that we observed in the pellet fraction. Copper ions seem to be unable to stimulate PrP interactions with the efficiency of Zn^2+^.

In conclusion, PrP^C^ differentiation based on metal binding provides a technique capable of distinguishing PrP^C^ isoforms bound within protein complexes. This may also enable the separation of full-length glycosylated and full-length nonglycosylated as well as truncated glycosylated isoforms, due to the decreased solubility of Zn^2+^ bound full-length glycosylated PrP^C^ in comparison to truncated PrP. Zn^2+^ selectively bound to full-length PrP^C^ but not to truncated and nonglycosylated PrP^C^ attributed to lack of the octarepeat sequence in the C1 fragment. As various PrP isoforms coexist in homogenized solutions, the separation of metal-bound and unbound PrP^C^ proteins based on different conformation and aggregation types can easily be accomplished by incorporating a high-speed centrifugation step.
